# Estimation of aerobic and anaerobic performance using a novel intermittent reactive agility test – An exploratory correlation study

**DOI:** 10.1371/journal.pone.0362462

**Published:** 2026-11-06

**Authors:** Bettina Karsten, Richard Latzel, Michael Kölling, Thorben Hülsdünker

**Affiliations:** 1 Institute for Lifecourse Development, School of Human Sciences, Centre for Exercise Activity and Rehabilitation, University of Greenwich, London, United Kingdom; 2 CBS University of Applied Sciences, Department of Health & Education, Cologne, Germany; 3 Faculty of Applied Natural Sciences and Industrial Engineering, Deggendorf Institute of Technology, Deggendorf, Germany; 4 Department of Sport, LUNEX, Differdange, Luxembourg; 5 Luxembourg Health & Sport Sciences Research Institute (LHSSRI), Differdange, Luxembourg; Eskisehir Technical University, TÜRKIYE

## Abstract

Separate tests are usually used to assess an athlete's ability to meet the aerobic and anaerobic demands of ball sports. These tests often compromise ecological validity by relying on linear runs and sprints rather than multidirectional reactive agility. This study investigated the correlations between a novel motor-cognitive reactive agility (RA) test and aerobic and anaerobic performance indices. Twenty-two trained participants ($\dot{\text{V}}$O_2max_: 56 ± 6.4 ml ∙ kg^-1^ ∙ min^-1^) performed a treadmill ramp test to determine maximum oxygen uptake ($\dot{\text{V}}$O_2_max), a running-based anaerobic sprint test (RAST) to determine peak power output (PPO), average power putput (POAV), fatigue index (FI) and anaerobic capacity (AC) and a motor-cognitive RA test on the SKILLCOURT. The RA test required participants to perform four 150 m maximal multidirectional change of directions runs with a break of 30 s. Running time, heart rate and power output were determined for all runs and correlated to $\dot{\text{V}}$O_2max_ and anaerobic performance indicators. Running time of the 4th SKILLCOURT run was correlated to $\dot{\text{V}}$O_2max_ (r_20_ = −0.80, p < 0.001) and a strong relation was found between PPO determined in the RAST and SKILLCOURT test (r_20_ = 0.72, p < 0.001). The SKILLCOURT RA test provides a promising tool for sport coaches to monitor aerobic and anaerobic performance indices, considering reactive agility. This needs to be validated in future studies.

## Introduction

The physical demands of intermittent sports involve high levels of aerobic and anaerobic energy turnover through repeated sprints, high speed running and change of direction (CoD), interspersed with short periods of lower intensity activity. This makes the aerobic [1] and anaerobic [2] capacities important contributing factors for athletes’ performance, rendering corresponding tests integral parts of performance diagnostics [3].

As the primary indicator of aerobic capacity, the maximal oxygen uptake (V̇O_2max_) is typically assessed on a treadmill with an incremental protocol until exhaustion. This is time-consuming, costly, and requires specialized equipment and personnel. For anaerobic testing, the Wingate test is often considered the gold-standard. It involves cycling at maximum effort for 30 seconds to determine anaerobic capacity (AC) and mechanical indices such as peak power output (PPO), average power output (POAV), and fatigue index (FI) [4]. However, both tests lack specificity and ecological validity for intermittent sports.

To address these limitations, Bangsbo et. al [5]. developed the Yo-Yo intermittent recovery test (Yo-YoIR) to assess aerobic performance. The test simulates intermittent sports with 2x20 m runs, a 180° turn, and 10 s active recovery (2x5 m). Speed increases stepwise and the test is performed until exhaustion [6]. The Yo-YoIR has two levels: Yo-YoIR1 and Yo-YoIR2, with the latter having a higher start speed and a faster speed ramping, and thereby comprising a greater contribution from the anaerobic pathway (4% and 10%, respectively) [7]. Based on the distance covered, the test estimates V̇O_2max_ [5], and it is considered valid and reliable [8,9]. Similarly, Draper and Whyte [10] translated the Wingate test into the running-based anaerobic sprint test (RAST), where athletes perform 6 x 35 m maximal sprints with 10 s active recovery. Like the Yo-YoIR, the RAST is deemed a valid and reliable alternative to the lab-based Wingate test. This has been confirmed for a wide spectrum of participants including healthy individuals [11], sprinters [12], male and female football players [13,14] as well as armed forces [15] with correlation coefficients between RAST and Wingate test typically in the range between r = 0.6–0.7. In the same vein, a recent systematic review and meta-analysis concluded that the RAST is comparable to the laboratory-based Wingate test for assessing anaerobic performance across various sports [16].

The Yo-YoIR and RAST are simple to use and better resemble the demands of intermittent sports. However, their ecological validity is still limited due to the missing reactive agility (RA) component. RA requires athletes to adapt their movements to external stimuli, which is essential for intermittent sports [17,18]. Importantly, CoD and RA are distinct abilities, each affected differently by fatigue [19]. As athletes in intermittent sports physically perform under cognitive load, this should be reflected in aerobic and anaerobic performance testing. While the Yo-YoIR test includes CoD, it involves pre-planned movements that differ from the reactive nature of sports. Additionally, CoD in the Yo-YoIR is limited to bidirectional 180° turns while research in basketball and football indicates that sport-specific actions also include 0–15° or 105–135° turns [20,21]. For instance, in football, > 80% of CoD movements are performed at angles <180° [22]. Similarly, linear runs at stepwise increasing speeds are not typical in these sports, where running actions are repeatedly performed at high or maximal intensity [23].

To address RA and multidirectional CoD movements, Born and colleagues [24] evaluated an incremental RA test based on the Yo-YoIR2 using the SpeedCourt technology. On a 5.25 m x 5.25 m floor with 12 contact plates participants completed a 40 m run with CoD movements based on visual cues between 0 and 180° followed by a 10 s rest. Speed was increased stepwise over the trials. While $\dot{\text{V}}$O_2_ in the SpeedCourt test correlated to the treadmill $\dot{\text{V}}$O_2max_ assessment (r = 0.59), the relation was lower compared to many studies using the YoYoIR tests [6]. Furthermore, the SpeedCourt also uses a stepwise speed increase that does not correspond to maximal sprints and CoD activities in ball sports.

In addition to these limitations in ecological validity of current tests, a feasibility perspective needs to be considered. Conducting both the Yo-Yo (aerobic) and RAST (anaerobic) for a comprehensive endurance performance assessment requires substantial time investment which limits their practical use.

Due to the higher relative contribution of lactic energy supply in the Yo-YoIR2, there have been attempts to correlate the YoYoIR tests to both aerobic and anaerobic performance. In a study conducting both tests, it was only the YoYoIR2 that was significantly correlated to PPO (r = −0.55) and FI (r = −0.66) but not POAV (r = −0.13) established in a Wingate test [25]. However, the correlation between YoYoIR2 and $\dot{\text{V}}$O_2max_ was only moderate (r = 0.53) and lower when compared to the YoYoIR1 which is typically around 0.7 [6]. Similarly, Krustrup et al. [26] observed a moderate relation between the YoYoIR2 and $\dot{\text{V}}$O_2max_ (r = 0.56) while there was no relation to a 5x30 m repeated sprint performance (r = 0.26). In the study of Thomas et al. [27], the YoYoIR2 did not attain a significant correlation with $\dot{\text{V}}$O_2max_ (r = 0.43). Together, these results indicate that neither the YoYoIR1 nor YoYoIR2 test provides a valid estimate of both aerobic and anaerobic performance.

A test overcoming the aforementioned drawbacks could assess the athletes' aerobic and anaerobic performance in a more ecologically valid and time-efficient way. The *motor-cognitive* reactive agility (RA) test on the SKILLCOURT (Skillcourt GmbH, Schweinfurt, Germany) has been developed for this purpose. The technology provides valid and reliable assessment of RA and motor-cognitive function [28,29]. It uses a 65” monitor and a 4 x 4 m court, where a LiDAR (light detection and ranging) continuously scans the athlete’s position. The RA test consists of four 150 m RA intervals with an intermittent 30 s active break. Distance, time, and heart rate (HR) are automatically tracked and analyzed. Intervals are performed as full RA sprints to 1) represent the multidirectional RA profile of the athlete in response to external stimuli and 2) replicate the all-out characteristics of the Wingate test or the RAST.

The present study evaluated the correlation between performance parameters established in the SKILLCOURT RA test and indices of aerobic ($\dot{\text{V}}$O_2max_) and anaerobic performance (PPO, POAV, FI, AC) derived from a treadmill $\dot{\text{V}}$O_2max_ test and RAST, respectively. Medium to strong correlations were expected between SKILLCOURT and both established tests (treadmill, RAST) that are comparable to the previously investigated YoYoIR and SpeedCourt tests.

## Materials and methods

### Sample size calculation

This study aimed to determine the quality of a motor-cognitive RA test on the SKILLCOURT technology for estimating aerobic and anaerobic performance as determined by a ramp-test on a treadmill and the RAST, respectively. A Pearson correlation of r = 0.56 corresponding to the relation between VO_2max_ and YoYo test performance [26] was used for sample size calculation. At an α-level of 0.05 and a minimum test power of 0.8 for a one-tailed test, this resulted in a minimum sample size of 16 participants. A dropout rate of 10% was expected, requiring 18 participants. With 26 participants and 22 in the final analysis, this study was sufficiently powered.

### Participants and ethics

26 healthy trained athletes corresponding to tier 2 according to the classification of McKay et al. [30] participated in this study. All participants were recruited from local sport clubs and contacted in verbal or written form. Three participants were not able to complete all tests due to illness and injury. One participant was excluded due to an exceptionally high resting-state lactate value (>2mmol∙l^-1^) in the $\dot{\text{V}}$O_2max_ test. The remaining 22 participants (17 males, 5 females, $\dot{\text{V}}$O_2max_: 56 ± 6.4 ml ∙ kg^-1^ ∙ min^-1^, age: 22.3 ± 2.4 yrs, height: 175.7 ± 8.8 cm, weight: 72.9 ± 10.4 kg) were considered. Athletes were recruited from ball and team sports (n = 15; football, handball, volleyball, basketball) and endurance sports (n = 7; running, cycling, swimming). In contrast to many previous approaches, this study recruited a mixed-gender sample and athletes from both ball/team sports and endurance sports for several reasons. First, as this is a novel test, including athletes from different sports provides higher external validity. Second, while ball/team sport athletes are more used to quick CoD movements corresponding to the characteristics of the new test as well as short sprints as reflected in the RAST, endurance athletes perform continuous exercises in line with the protocol of the treadmill test. If the test provides good association with established protocols despite this additional layer of variability, it supports the test’s robustness against differences in energetic and movement profiles between sports (ball/team vs. endurance). Finally, participants were carefully selected to ensure that overall group variability in VO_2max_ (±6.4 ml ∙ kg^-1^ ∙ min^-1^) in our sample corresponds to previous research. In fact, variability did not significantly differ from five previous studies assessing the validity of the YoYo test ranging from 4.3 [24] to 6.9 ml ∙ kg^-1^ ∙ min^-1^ [9]. Therefore, including both ball/team sport athletes and endurance athletes did not result in an exceptionally high variability when compared to previous studies that recruited participants from a single sport or gender. This is important, as a higher variability may increase the likelihood of finding a significant correlation. Participants had a weekly training load of 2.2 (±1.4) sessions in their sport and 2.9 (±1.2) sessions assigned to associated content such as strength and conditioning training. Athletes had to refrain from caffeine 4 h prior to each test and intense physical exercise one day before the test days. All participants were informed about the experimental protocol, and their written consent was obtained. The recruitment period started on the 05/02/2024 and ended on the 03/05/2024. The study was approved by the Luxembourgish national research ethics committee in accordance with the Declaration of Helsinki (202207/01 v2.0).

### Experimental protocol

Participants visited the lab on three test days separated by at least 48 hours. Tests were conducted at the same time of the day (±2 h) to avoid circadian rhythm effects. The three test days were assigned to an incremental treadmill ramp test, the RAST and the motor-cognitive RA test on the SKILLCOURT. The order of tests was randomized. All tests started with a 5 min resting period after which HR and lactate were measured. The incremental ramp-test on the treadmill (Quasar, h/p/cosmos sports & medical GmbH, Nussdorf-Traunstein, Germany) followed the protocol of Maier et al. [31]. Ventilation was continuously recorded breath-by-breath using a Meta Max 3B spiroergometry (Cortex Biophysics GmbH, Leipzig, Germany). Participants started with a constant-speed warm-up phase at a velocity of 6 km ∙ h^-1^ at 1% inclination for 3 minutes. This was followed by the ramp protocol where the velocity was increased every 30 s by 0.5 km ∙ h^-1^ and at an incline of 7% (Fig. 1A). Participants were instructed to run as long as possible. The test finished at full exhaustion when participants left the running belt. Lactate and maximal HR were determined immediately after the end of the test which was followed by a cool-down at 4 km ∙ h^-1^ for 3 min.

**Fig 1:**
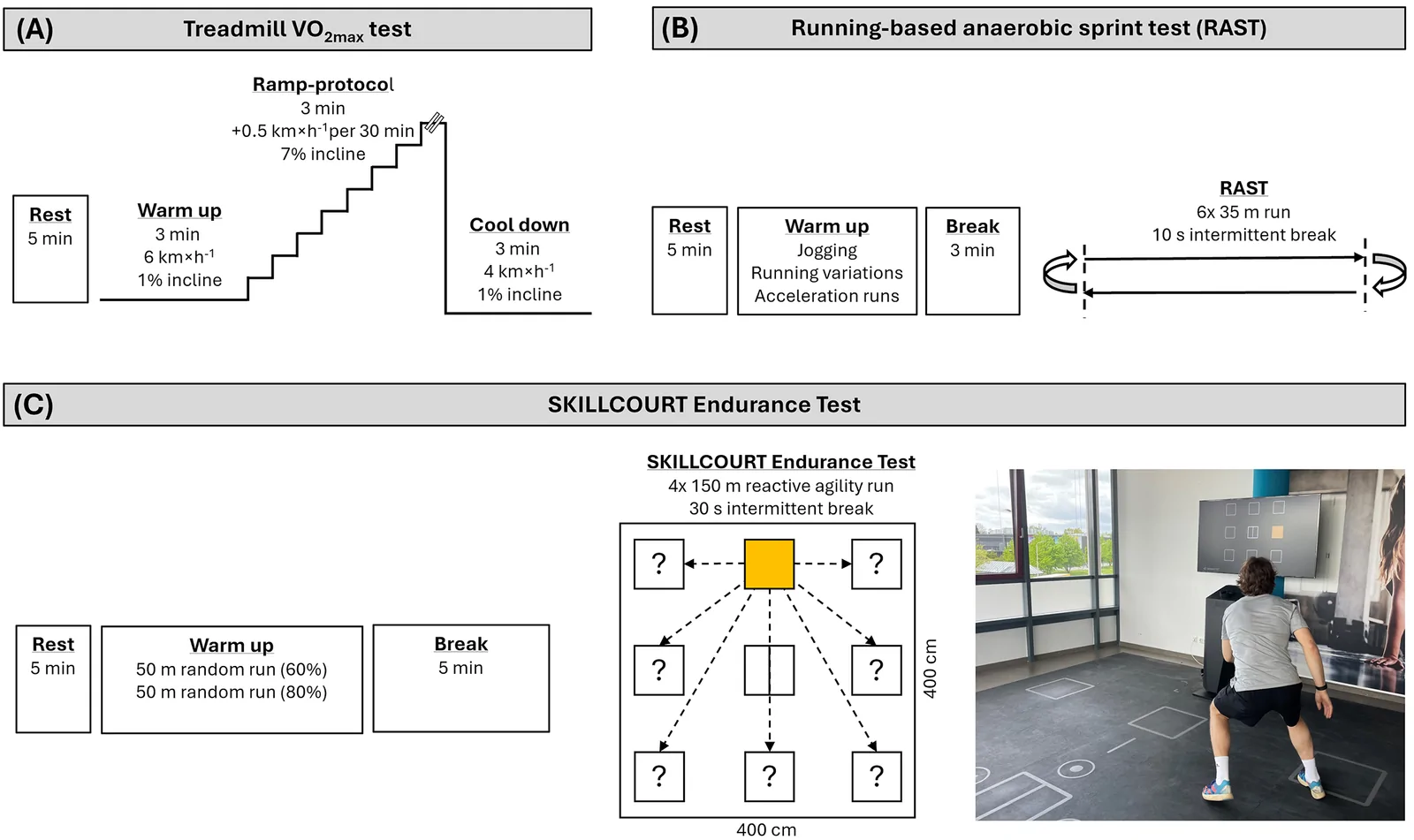
Graphical illustration of the testing protocol A) $\dot{𝐕}$O_2max_ test, B) running-based anaerobic sprint test and C) SKILLCOURT RA test.

The RAST was conducted outdoors on a running track and based on the work of Draper and Whyte [10] which has been shown a valid and reliable field test alternative to the Wingate test on a cycle ergometer [15,32]. Participants started with a standardized warm-up including jogging, running variations and four acceleration runs at 80% of maximum performance. This was followed by a ~ 3-min rest period for recovery. Participants performed 6 maximal linear sprints over a distance of 35 m with a 10 s break in between. The experimenter provided a countdown over the last 5 s prior to the start of the next run (Fig 1B). Participants were instructed to come to a full stop at the starting line before the next run started. Following the test, HR and lactate were determined. Sprinting times were measured using timing gates (WITTY, Microgate, Bozen, Italy).

For the RA test, the SKILLCOURT technology was used. Participants started with a standardized warm-up including two 50-m reactive agility runs with a 5–7 min stretching and mobilization session in between to get prepared for the change of direction movements (Fig 1C). The 50 m reactive agility runs were performed on the SKILLCOURT resembling the test design in the RA test. Participants were presented with an illustration of the eight outer target fields of the SKILLCOURT of which one was highlighted in yellow. When the highlighted field was reached as detected by the LiDAR scanner, the next field was highlighted. The order of fields was fully randomised with the constraint that the same field could not be highlighted twice consecutively, as this would correspond to a covered distance of 0 m. The running sequence was generated dynamically during each run until 150 m were reached. Distance was calculated based on the distance between target fields and stopped after the defined distance was reached. Consequently, participants could neither anticipate nor learn the running pattern, and the number of highlighted fields could vary between runs dependent on the randomization sequence. Participants were instructed to perform the first 50 m warm up trial at about 60% or their maximum, the second warm-up trial at about 80% of their maximum to familiarize with the SKILLCOURT. Following the warm-up, participants had a break of 5 min. The SKILLCOURT RA test was composed of four 150 m maximal reactive agility runs with an intermittent break of 30 s. The protocol was run automatically by the system and started with a 3-s countdown. Participants were instructed to perform the task as fast as possible. During the 30 s break, participants were allowed to move on the court but not run. At the latest 5 s before the next run, participants had to enter the center field of the SKILLCOURT for a standardized starting position.

### Data analysis

The $\dot{\text{V}}$O_2max_ as the primary outcome parameter for the treadmill test was defined as the highest $\dot{\text{V}}$O_2_ within a 30-s interval during the incremental test. A valid $\dot{\text{V}}$O_2max_ was assumed when a levelling off in $\dot{\text{V}}$O_2_ (< 150 mL ⋅ min^-1^) occurred despite an increase in treadmill speed. In case of no $\dot{\text{V}}$O_2_ plateau, secondary criteria such as RER ≥ 1.1 and blood [lactate]  ≥ 8 mmol·L^-1^ were used [33]. For each run of the RAST, the PO (body mass ∙ distance² ∙ time^-3^) was calculated. Parameters derived from the RAST include PPO, POAV, FI ($FI=\frac{\left(PPO-POmin\right)}{totaltime(s)}$) and AC as the sum of all six PO. For the SKILLCOURT test the running time for each trial as well as the overall running time was included in the data analysis. As in the RAST, PO was calculated based on the running times and BW which formed the basis for determining PPO, POAV, FI and AC for the SKILLCOURT test. In addition, the HR was determined at the end of each run. The recovery HR was measured 60 s after the last run.

### Statistics

Statistical analyses were conducted using the JASP software package. All parameters of interest were assessed for normality using the Shapiro–Wilk test. Non-parametric tests were applied when the assumption of normality was violated. Accordingly, lactate data were analyzed using a Friedman test. All other variables showed no significant deviation from normality in either the full sample or the subgroups; therefore, parametric repeated-measures ANOVAs (RM-ANOVA) and Pearson correlation analyses were applied. Control analyses (Friedman test/RM-ANOVA followed by Bonferroni-corrected post-hoc tests) were performed for resting state lactate and HR values to test for baseline differences between test days. To determine the relation between motor-cognitive performance in the RA test and aerobic performance, behavioural (running time) and physiological (HR and lactate) parameters obtained on the SKILLCOURT were correlated to the $\dot{\text{V}}$O_2max_ derived from the ramp test. A Bland-Altman agreement analysis was not performed because VO_2max_ was not directly measured during the SKILLCOURT test. Therefore, the present study assesses associations between behavioural SKILLCOURT performance and treadmill-measured VO_2max_, rather than agreement between two measurements of the same variable. Similarly, behavioural performance metrics in the SKILLCOURT RA test were correlated to indices of anaerobic capacity derived from the RAST. For each dependent variable, p-values were Bonferroni-corrected for multiple correlations. Linear regression models were calculated for the parameter showing the strongest correlation per dependent variable (VO_2max,_ PPO, POAV, AC, FI). As an exploratory approach, correlations were further calculated for ball and team sport athletes (n = 15) and endurance athletes (n = 7) separately. Correlations were considered small (<0.3), moderate (<0.5), large (<0.7), very large (<0.9) and nearly perfect (>0.89). For all correlation analyses, 95% confidence intervals have been calculated. The significance threshold was set to p < 0.05.

## Results

ANOVA comparisons revealed a significant difference between conditions in resting HR (F_2,42_ = 3.55, p = 0.038, η_p_^2^ = 0.144) indicating a lower HR before the RAST when compared to the RA test and treadmill test. However, post-hoc tests did not reach significance (p ≥ 0.07, d ≤ 0.57). The control analysis for lactate was not significant (Χ² = 0.72, p = 0.7, Kendall’s W: 0.016).

### SKILLCOURT RA test characteristics

The SKILLCOURT RA test had an effective running time across all four intervals of 357 s (±24.9 s). Together with the fixed active recovery time of 90 s (3 x 30 s), this resulted in a test duration of 447 s corresponding to about 7.5 min. Test duration for the treadmill test was 693 s (±87 s) corresponding to about 11.5 minutes. The maximum HR during the SKILLCOURT RA test was 185 (±8) bpm which was not significantly different from the $\dot{\text{V}}$O_2max_ test (p = 0.490, d = 0.294) but higher than in the RAST (p = 0.028, d = 0.401). Lactate values reached immediately after the last run were 10.2 mmol×l^-1^ which was at the same level as the $\dot{\text{V}}$O_2max_ test (p = 0.566, d = 0.34) but significantly higher when compared to the RAST (p = 0.008, d = 0.689) (Table 1).

**Table 1:** Summary of variables derived from the SKILLCOURT RA test, treadmill ramp test and RAST expressed as mean (±standard deviation). Number of participants = 22.

Test	Parameter	Average (±standard deviation)
**SKILLCOURT RA test**	Pre_HR (bpm)	85 (±11.5)
	Post_HR (bpm)	185 (±8.0)
	Pre-lactate (mmol∙l^−1^)	0.9 (±0.3)
	Post-lactate (mmol∙l^−1^)	10.2 (±3.4)
	Test duration (min:ss)	7:27 (±0:25)
	Run 1 time (s)	85 (±6.9)
	Run 2 time (s)	89 (±6.3)
	Run 3 time (s)	90 (±7.5)
	Run 4 time (s)	91 (±7.1)
	Overall run time (s)	357 (±24.9)
	Peak power output (W)	2.8 (±0.7)
	Average power output (W)	2.2 (±0.4)
	Anaerobic capacity (W)	9.7 (±2.2)
	Fatigue index (W ∙ s^−1^)	0.002 (±0.001)
	Heart rate run 1 (bpm)	165 (±13.1)
	Heart rate run 2 (bpm)	170 (±10.8)
	Heart rate run 3 (bpm)	172 (±10.6)
	Heart rate run 4 (bpm)	183 (±9.0)
	Recovery heart rate (bpm)	140 (±14.8)
**Treadmill VO**_**2max**_ **test**	Pre_HR (bpm)	85 (±12.4)
	Post_HR (bpm)	187 (± 8.9)
	Pre-lactate (mmol∙l^−1^)	0.9 (±0.3)
	Post-lactate (mmol∙l^−1^)	9.1 (±3.2)
	Test duration (min:ss)	11:33 (±1:27)
	VO_2max_ (ml ∙ kg^-1^ ∙ min^−1^)	56 (±6.4)
**RAST**	Pre_HR (bpm)	79 (±12.3)
	Post_HR (bpm)	181 (±9.8)
	Pre-lactate (mmol∙l^−1^)	0.90 (±0.2)
	Post-lactate (mmol∙l^−1^)	8.1 (±2.7)
	Test duration (min:ss)	1:25 (±00:03)
	Peak power output (PPO) (W)	611 (±189)
	Average power output (POAV) (W)	472 (±113)
	Anaerobic capacity (AC) (W)	2835 (±682)
	Fatigue index (FI) (W ∙ s^−1^)	6.5 (±3.7)

RA = reactive agility, VO_2max_ = maximum oxygen uptake, HR = heart rate

### VO_2max_

Significant correlations between $\dot{\text{V}}$O_2max_ and behavioural outcomes on the SKILLCOURT were observed for running time in runs 3 and 4 (Fig 2A) as well as the overall running time (r_20_ ≥ −0.65, p ≤ 0.045). For physiological parameters including HR at the end of the runs as well as recovery HR 60 s after the last run, no correlation reached the significance level (r_20_ ≤ −0.49, p ≥ 0.100). $\dot{\text{V}}$O_2max_ was best correlated to the running time in run 4 (r_20_ = −0.80 [95% CI: −0.57, −0.91], p < 0.001). The linear regression analysis revealed an adjusted r^2^ of 0.62 (F_1,20_ = 35.50, p < 0.001, standardized coefficient: −0.79, SEE: 3.93). Exploratory subgroup analyses showed similar patterns of correlation when only ball and team sport players were considered (r_13_ = −0.79 [95% CI: −0.46, −0.93], p < 0.001). Similarly, there was a strong correlation also for the small sample of endurance athletes (r_5_ = −0.92 [95% CI: −0.53, −0.99], p = 0.003).

**Fig 2:**
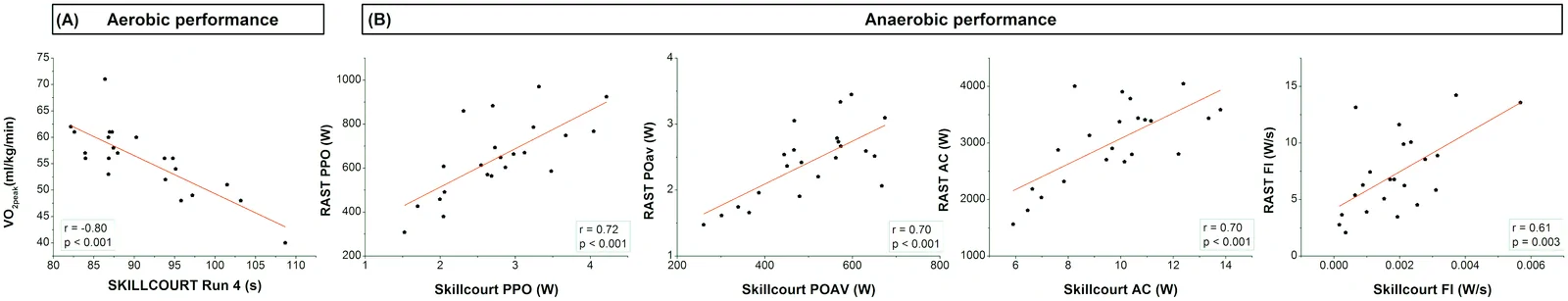
Correlations between A) SKILLCOURT RA test and $\dot{𝐕}$O_2max_ result and B) SKILLCOURT RA test and anaerobic performance indices. Note: The low peak power values for the Skillcourt RA test are related to the equation for the power calculation ($\frac{bodymass(kg)*distance(m{)}^{\text{2}}}{{time(s)}^{\text{3}}}$). As due to the change of direction movement on the SKILLCOURT, time disproportionately increases with distance when compared to straight running as in the RAST. Consequently, the power value drops.

### RAST

Indicators of anaerobic performance calculated from the RAST were correlated against the equivalent measures of the SKILLCOURT test (Fig 2B). PPO obtained from the RAST and SKILLCOURT significantly correlated at a high effect size (r_20_ = 0.72 [95% CI: 0.42, 0.87], p < 0.001). The linear regression revealed an adjusted r^2^ of 0.50 (F_1,20_ = 21.09, p < 0.001, standardized coefficient: 0.72, SEE: 125.29). When limiting the analysis to ball/team sport athletes, a comparably high correlation of r_13_ = 0.70 [95% CI: 0.28, 0.89] was observed (p = 0.004). For endurance athletes a correlation of r_5_ = 0.85 [95% CI: 0.27, 0.98] was observed (p = 0.016). Significant correlations across participants were further observed for POAV (r_20_ = 0.70 [95% CI: 0.39, 0.87], p < 0.001), AC (r_20_ = 0.70 [95% CI: 0.39, 0.87], p < 0.001) and FI (r_20_ = 0.61 [95% CI: 0.25, 0.82], p = 0.003). The corresponding linear regression models revealed for POAV an adjusted r² of 0.46 (F_1,20_ = 18.95, p < 0.001, standardized coefficient: 0.70, SEE: 86.9), for AC an adjusted r² = 0.49 (F_1,20_ = 18.95, p < 0.001, standardized coefficient: 0.70, SEE: 521.5) and for FI an adjusted r² of 0.34 (F_1,20_ = 11.79, p = 0.003, standardized coefficient: 0.61, SEE: 2.89). HR and recovery HR measures did not correlate to any of the parameters of interest in the RAST (r_20_ ≤ 0.40, p ≥ 0.315).

As the calculation of power output for RAST and SKILLCOURT shared the body-mass term which might result in spurious correlations, control analyses on raw running times as well as partial correlations were conducted. For the first run in the RAST and SKILLCOURT forming the basis for the PPO calculation a correlation of r = 0.68 (p < 0.001, [95% CI: 0.35, 0.85]) was observed. For the overall running time the correlation reached r = 0.72 (p < 0.001, [95% CI: 0.43, 0.88]). Partial correlations accounting for body mass all remained significant for PPO (r = 0.62, p = 0.003), POAV (r = 0.61, p = 0.004) and AC (r = 0.61, p = 0.004).

## Discussion

Confirming our hypothesis, the main findings of the present study were that test performance in the SKILLCOURT RA test was related to $\dot{\text{V}}$O_2max_ determined in a treadmill ramp test and to indicators of anaerobic performance (PPO, POAV, AC, FI) derived from the RAST.

As the test has been developed as an ecologically valid alternative to existing field-based endurance tests, the results must be interpreted in relation to the Yo-Yo intermittent recovery test which is the most frequently used alternative to lab-based treadmill tests for assessing aerobic performance in ball and team sports such as football [3]. For the Yo-YoIR test, correlations typically range between moderate [9,25,26] to high [27] ranging between r = 0.56–0.87.

These previously reported correlations between lab-based, directly measured $\dot{\text{V}}$O_2max_ and estimations of $\dot{\text{V}}$O_2max_ from Yo-YoIR are comparable to or even lower when compared to the correlation between direct $\dot{\text{V}}$O_2max_ and estimation from SKILLCOURT RA test presented here (r = 0.80). These initial results support the suggestion that the RA test may provide a promising protocol for estimating aerobic performance which however requires validation in future studies. To gain valid and reliable results, field-based assessments such as the YoYoIR tests require experienced staff with at least two raters and supported by additional technology such as timing gates and gyroscopes [6]. This compromises the objectivity of the test and requires human resources, limiting its applicability in professional settings. The LiDAR-based distance and time measurement and automatic data analysis can provide a more objective, standardized and time efficient alternative. Further, the task design in the RA test using a maximal RA protocol, rather than straight runs with single 180° turns at linearly increasing speed may better reflect the actual demands during ball and team sports [20].

When compared to a first attempt of using multidirectional reactive agility runs on the SpeedCourt to estimate aerobic performance [24], the correlation to $\dot{\text{V}}$O_2max_ was substantially higher for the RA test although a direct comparison between correlation coefficients slightly failed to reach the significance level (r = 0.8 vs. r = 0.59, p = 0.087, Cohen’s q = 0.47). Importantly, strong correlations were observed in both ball and team sport athletes as well as in endurance athletes. These findings suggest that the test may be applicable across different sports although there was a strong effect for the difference in correlations between groups that however remained non-significant (p = 0.17). Further, the small sample size of endurance athletes (n = 7) and heterogeneous sample in general warrants cautious interpretation of these findings, which should be validated in future studies.

In addition to aerobic capacity, performance in the SKILLCOURT RA test was strongly related (r = 0.61–0.72) to indicators of anaerobic performance derived from the RAST. This conclusion remained unchanged when controlling for body mass, which enters both power calculations identically. Correlations were only slightly reduced by no more than 0.10 when using raw running times or partial correlations and remained highly significant. The RAST is an established test for determining anaerobic performance in ball and team sports as an alternative to the cycling-based Wingate test [14,15]. While there is less research on anaerobic performance, previous studies using the Yo-Yo test assessed its relation to both aerobic (VO_2max_) and anaerobic performance. Karakoc et al. [25] observed a significant negative correlation between PPO (r = −0.55) and FI (r = −0.66) but not POAV (r = -0.13) measured in a Wingate test for the YoYoIR2 in trained football players ($\dot{\text{V}}$O_2max_: 59.95 ml ∙ kg^-1^ ∙ min^-1^). However, no correlations were found for the YoYoIR1 test [25]. The researchers consequently recommended the YoYoIR2 as more suitable for tracking aerobic and anaerobic performance. This is in line with the suggestion that the YoYoIR1 places more emphasis on aerobic endurance performance, whereas the YoYoIR2 is designed to include a greater contribution from anaerobic energy sources [5,6]. Further, and somewhat in contrast to the results of Karakoc et al. [25], Krustrup et al. [26] observed no relationship (r = 0.26) between the YoYoIR2 and 5x30 m sprint performance with 25 s active recovery in a group of recreational athletes. These findings indicate that the Yo-Yo test is not suitable for estimating anaerobic performance which may be related to the test setup. For the accurate estimation of anaerobic indices, both the Wingate and the RAST necessitate all-out efforts. This, by definition, requires the depletion of Wprime, which is located in the severe-intensity domain, and it is defined as the finite amount of work that can be performed above the maximum aerobic metabolic rate without loss of homeostasis [34]. The repeated all-out RA sprints in the SKILLCOURT RA test better resemble these demands which can explain the consistently high and significant correlation to the RAST across all parameters (r ≥ 0.61).

Together, these results indicate that performance in the 4^th^ run of the SKILLCOURT RA test may provide a promising estimate of aerobic performance that is comparable to or even higher when compared to established tests such as the Yo-YoIR. Similarly, estimates of peak power are related to established anaerobic performance measures from the RAST. The pilot results of this study suggest that the RA test may have the potential as a time-efficient single test for estimating both aerobic and anaerobic performance in an objective, standardized and sport-specific motor-cognitive environment. However, these first results on the RA protocol require further investigation on validity, reliability, usefulness and ecological validity. This also applies to the sub-group analyses where especially for the endurance athletes, the sample was very small (n = 7).

### Practical application

The newly developed RA test using the SKILLCOURT technology provides promising pilot results for estimating aerobic and anaerobic performance. Prospectively, and following additional validation it may thus provide a more sport-specific, time efficient and objective alternative to established sport-specific aerobic and anaerobic performance tests such as Yo-Yo or RAST. This applies to both team ball sport and endurance athletes although for the endurance athlete group the sample was very small (n = 7). The all-out RA runs better resemble the motor-cognitive demands of ball and team sports and objective performance tracking via optical LiDAR technology allows for a standardized and objective test performance with low staff demands. Further, estimating both aerobic and anaerobic performance in a single test provides a time efficient assessment of different metabolic pathways relevant in sports. Therefore, these preliminary results on the RA test may provide the potential of its integration as an aerobic and anaerobic assessment for performance diagnostics in team ball sports. However, further research confirming the results and analyses into the reliability and ecological validity of the test is needed to support its usefulness in sport.

### Limitations

While this study indicates that the SKILLCOURT RA test provides promising results for determining aerobic and anaerobic performance, it must be considered that this study was designed to provide a first proof-of-concept using a correlation analysis. Follow-up projects are needed to address the test-retest reliability of the RA protocol. In the present study this was an unrealistic ambition as between-day analyses would have required at least six visits to the laboratory per participant. To support the test’s usefulness, longitudinal studies on the sensitivity to identify training-induced changes in aerobic and anaerobic performance are required. Further, studies with larger and more homogeneous samples of ball/team sport athletes and endurance athletes as well as athletes from different disciplines can provide additional evidence for the test’s usefulness in practical application. The same applies to the investigation of potential differences between males and females. As the sample size was small for the subgroups (team ball sport vs. endurance sport and males vs. females) no definitive conclusions can be drawn on the generalizability of the results which need to be confirmed in follow-up projects. Especially for endurance sports, the very small sample limits the generalizability for this population. Moreover, as the RA test performance was related to both aerobic and anaerobic performance indices, future studies with larger samples should further evaluate multiple regression models including aerobic and anaerobic parameters to determine their relative contribution to the RA test performance. To support the ecological validity of the novel test, correlation analyses with in-game performance metrics determined by GPS such as running distance or number of sprints are required. As the level of training appears to play a major role in the test results, they need to be confirmed in athlete populations. Longitudinal training studies including the novel protocol and determining its sensitivity to performance changes in VO_2max_ are further needed to support its usefulness. Finally, the study did not directly measure $\dot{\text{V}}$O_2max_ on the SKILLCOURT, which represents a critical methodological limitation. As a result, the analysis is confined to examining correlations rather than true agreement, preventing any definitive conclusions about the validity or interchangeability of the measurements obtained. To enable an agreement analysis, future studies should measure oxygen uptake during the RA test to determine whether the protocol elicits a true VO_2max_ response. It should also be noted that the regression analyses were exploratory and were not independently cross-validated. Therefore, the reported models and SEE values should not be interpreted as validated predictive equations.

## Conclusions

To the best of our knowledge, no study to date has investigated estimates of both aerobic and anaerobic performance within a single reactive agility test. Our pilot results show significant correlations for both whilst considering RA, which is essential in ball and team sports. The SKILLCOURT RA test shows promising associations with established measures of aerobic and anaerobic performance and may warrant further investigation as a sport-specific assessment approach. However, future studies must further examine the test’s reliability, explore its relationship with match performance, and investigate the underlying physiological mechanisms to support its validity and practical utility in sports.
